# Passengers' destinations from China: low risk of Novel Coronavirus (2019-nCoV) transmission into Africa and South America

**DOI:** 10.1017/S0950268820000424

**Published:** 2020-02-26

**Authors:** Najmul Haider, Alexei Yavlinsky, David Simons, Abdinasir Yusuf Osman, Francine Ntoumi, Alimuddin Zumla, Richard Kock

**Affiliations:** 1 The Royal Veterinary College, University of London, Hawkshead Lane, North Mymms, Hatfield, Hertfordshire; 2 Institute of Health Informatics, University College London, London, UK; 3 Fondation Congolaise pour la Recherche Médicale (FCRM), Brazzaville, Republic of Congo; 4 Institute for Tropical Medicine/University of Tübingen, Tubingen, Germany; 5 Department of Infection, Division of Infection and Immunity, UCL Centre for Clinical Microbiology, Royal Free campus, London, UK; 6 NIHR Biomedical Research Centre, UCL Hospitals NHS Foundation Trust, London, UK

**Keywords:** 2019-nCoV, Africa, China, risk map, transmission, Wuhan, COVID-19, SARS-COV-2

## Abstract

Novel Coronavirus (2019-nCoV [SARS-COV-2]) was detected in humans during the last week of December 2019 at Wuhan city in China, and caused 24 554 cases in 27 countries and territories as of 5 February 2020. The objective of this study was to estimate the risk of transmission of 2019-nCoV through human passenger air flight from four major cities of China (Wuhan, Beijing, Shanghai and Guangzhou) to the passengers' destination countries. We extracted the weekly simulated passengers' end destination data for the period of 1–31 January 2020 from FLIRT, an online air travel dataset that uses information from 800 airlines to show the direct flight and passengers' end destination. We estimated a risk index of 2019-nCoV transmission based on the number of travellers to destination countries, weighted by the number of confirmed cases of the departed city reported by the World Health Organization (WHO). We ranked each country based on the risk index in four quantiles (4^th^ quantile being the highest risk and 1^st^ quantile being the lowest risk). During the period, 388 287 passengers were destined for 1297 airports in 168 countries or territories across the world. The risk index of 2019-nCoV among the countries had a very high correlation with the WHO-reported confirmed cases (0.97). According to our risk score classification, of the countries that reported at least one Coronavirus-infected pneumonia (COVID-19) case as of 5 February 2020, 24 countries were in the 4^th^ quantile of the risk index, two in the 3^rd^ quantile, one in the 2^nd^ quantile and none in the 1^st^ quantile. Outside China, countries with a higher risk of 2019-nCoV transmission are Thailand, Cambodia, Malaysia, Canada and the USA, all of which reported at least one case. In pan-Europe, UK, France, Russia, Germany and Italy; in North America, USA and Canada; in Oceania, Australia had high risk, all of them reported at least one case. In Africa and South America, the risk of transmission is very low with Ethiopia, South Africa, Egypt, Mauritius and Brazil showing a similar risk of transmission compared to the risk of any of the countries where at least one case is detected. The risk of transmission on 31 January 2020 was very high in neighbouring Asian countries, followed by Europe (UK, France, Russia and Germany), Oceania (Australia) and North America (USA and Canada). Increased public health response including early case recognition, isolation of identified case, contract tracing and targeted airport screening, public awareness and vigilance of health workers will help mitigate the force of further spread to naïve countries.

## Introduction

On 31 December 2019, local hospitals in Wuhan, China reported that they had detected a series of cases of Novel Coronavirus-infected pneumonia to the World Health Organization (WHO) [1]. On 7 January, the causative agent was identified by the Chinese Centre for Disease Control and Prevention as a Novel Coronavirus and designated ‘2019-nCoV’ and finally as "SARS-COV-2". Epidemiological investigations identified the local Huanan seafood wet market as the location of an initial exposure event [2]. The market was closed on 31 December 2019 [2, 3] and wildlife market activity was banned countrywide. Despite travel restrictions to and from the city imposed by Chinese authorities to limit the potential dispersion of the virus beyond the region [4, 5], international cases continue to be reported.

As of 5 February 2020, there were 24 554 confirmed Novel Coronavirus-infected pneumonia (COVID-19) cases in 27 countries or territories, of which 24 363 (99.2%) were within mainland China [6]. The locations of internationally imported cases are consistent with risk models generated from flight data out of Wuhan city. Transmission from mildly symptomatic (i.e. cough, lethargy, myalgia) infected individuals was identified early in the course of this outbreak, with human-to-human transmission detected in international case series [7].

The timing of this outbreak around the lunar new year widely celebrated in China coincides with a period of highest annual human movement patterns in the region and between China and globally [8], increasing the potential for rapid geographic dispersal of the infection. Further, recent investment in the African continent by the Chinese state and private investors has led to an increasing Chinese diaspora [9] and a greater number of direct and indirect flight connections to the African continent from China [10].

There are few studies available on global risk of 2019-nCoV spread [4, 5, 11]. Bogoch *et al*. [5] and Chinazzi *et al*. [11] estimated the risk of importation of 2019-nCoV from major Chinese cities to the most frequent international destinations. Wu *et al*. estimated the risk of international spread compared to domestic outbound flights [4]. These articles do not model the cumulative risk of importation of 2019-nCoV in a country and instead focus on specific points of entry. Here, we considered all the end destinations of flights from four important cities of China involving 168 countries/territories around the world and calculated the total risk of transmission into a country by aggregating the risk associated with all the entry airports of the country. We further looked in more detail at the risk to Africa where the health infrastructure would be challenged tracking a new epidemic across its 54 countries.

The aim of the current study was to explore the effect of sustained transmission from the four Chinese cities of Wuhan, Beijing, Shanghai and Guangzhou on international disease importation risk to 168 countries and territories, with a specific focus on Africa where current levels of healthcare infrastructure could provide a significant challenge for managing this novel epidemic.

## Methods

### Data

We extracted modelled flight data for the final destination of passengers travelling from four Chinese cities (including domestic and international destinations) from the FLIRT database [12, 13]. FLIRT was designed to predict the flow and likely destination of infected travellers through the air travel network. It uses a database of flight schedules from over 800 airlines and displays direct flight connections in addition to a modelled end destination (three-letter IATA code for airports). Flight connection data and passenger numbers are based on the data collected since October 2014 [12]. We extracted the simulated passenger's data for each week for the period of 1 January to 31 January 2020 from four major Chinese cities: Wuhan, Beijing, Shanghai and Guangzhou. The simulation can process up to 20 000 passengers' information for a particular time frame from any city (including surrounding airports). We collected the reported 2019-nCoV case data from the WHO's daily situation update website [14].

### Estimation of risk of transmission

To estimate the relative risk of 2019-nCoV transmission, we considered all infected passengers who travelled between 1 January and 31 January to possess a maximum risk of transmission 1 (and no infected passengers means no risk) and estimated the relative risk of each country based on the number of passengers who travelled from each of the four cities. Thus countries with a higher number of passengers travelling from any of these cities had a higher risk of transmission. We then weighted the risk estimated for each city with the number of reported infected people in each city by 31 January 2020 [14] and estimated the mean average risk of transmission termed as ‘Risk index’ which follows the equation below:
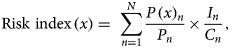
where *x* is the destination country, Risk index (*x*) is the risk of infection importation in country *x*, *P*(*x*)_*n*_ is the number of passengers to country *x* from city *n*, *P*_*n*_ is the total number of passengers who left city *n*, *I*_*n*_ is the number of infected people in city *n* and *C*_*n*_ is the population size of city *n*. The risk index denotes the risk of at least one case being imported into a country or territory where 1 means an absolute certainty and 0 means no risk at all. Our model assumed that there is no case outside China and thus ignored if any country already had imported case(s). In countries where 2019-nCoV is already detected, the risk index would explain the risk of importing additional infected individuals from China.

We performed a Pearson correlation coefficient test between the risk index of the country and the WHO's reported case number from the country. We grouped the countries in four quantiles based on the risk index where high-risk countries were grouped as the 4^th^ (>75^th^ percentiles) and the 3^rd^ (>50^th^ to ⩽75^th^ percentiles) quantiles and low-risk countries were grouped as the 2^nd^ (>25^th^ to ⩽50^th^ percentiles) and the 1^st^ (⩽25^th^ percentile) quantiles (Table 1).

**Table 1.** The list of countries or territories based on their risk index in different quantiles for 2019-nCoV (SARS-COV-2) transmission

	4^th^ Quantile risk index (highest risk)		3^rd^ Quantile risk index		2^nd^ Quantile risk index		1^st^ Quantile risk index (lowest risk)	
Sl/Rank	Country	Risk index	Country	Risk index	Country	Risk index	Country	Risk index
1	China	0.609603126	Sweden	0.000320248	Kenya	3.53 × 10^−05^	Jamaica	3.84 × 10^−06^
2	Thailand	0.099432816	Laos	0.000296262	Peru	3.18 × 10^−05^	Serbia	3.22 × 10^−06^
3	Cambodia	0.05294058	Brazil	0.00027186	Algeria	3.11 × 10^−05^	Togo	2.73 × 10^−06^
4	Malaysia	0.041899039	Denmark	0.000254904	French Polynesia	3.07 × 10^−05^	Uganda	2.71 × 10^−06^
5	Canada	0.02730388	Oman	0.000248884	Iceland	3.00 × 10^−05^	Tonga	2.69 × 10^−06^
6	USA	0.021169936	Israel	0.000221865	Samoa	2.74 × 10^−05^	The Bahamas	2.39 × 10^−06^
7	Japan	0.01479856	Ukraine	0.000209515	Tanzania	2.73 × 10^−05^	Cote d'Ivoire	2.02 × 10^−06^
8	India	0.010256629	Poland	0.0002	Palau	2.63 × 10^−05^	Suriname	2.01 × 10^−06^
9	UK	0.008786839	Brunei	0.000181028	Djibouti	2.45 × 10^−05^	Vanuatu	1.99 × 10^−06^
10	South Korea	0.008072566	Czech Republic	0.000179806	Belarus	2.40 × 10^−05^	Albania	1.90 × 10^−06^
11	Vietnam	0.007928803	Northern Mariana Islands	0.000177972	Bosnia and Herzegovina	2.40 × 10^−05^	Malta	1.73 × 10^−06^
12	Singapore	0.007784474	Belgium	0.000175566	Cook Islands	2.26 × 10^−05^	Guinea	1.60 × 10^−06^
13	Hong Kong	0.007636714	Maldives	0.000172453	Colombia	1.94 × 10^−05^	Namibia	1.55 × 10^−06^
14	Indonesia	0.007197131	Norway	0.000171441	Papua New Guinea	1.88 × 10^−05^	Democratic Republic of Congo	1.41 × 10^−06^
15	United Arab Emirates	0.007089607	Kuwait	0.000166929	Nigeria	1.85 × 10^−05^	Rwanda	1.22 × 10^−06^
16	France	0.006814962	Egypt	0.000139373	Jordan	1.83 × 10^−05^	Honduras	1.02 × 10^−06^
17	Turkey	0.00568808	Iran	0.000137181	Cuba	1.79 × 10^−05^	Gabon	8.98 × 10^−07^
18	Australia	0.005671745	Mongolia	0.000132319	Argentina	1.70 × 10^−05^	Republic of Congo	7.22 × 10^−07^
19	Russia	0.005592859	Chile	0.000129975	Tunisia	1.65 × 10^−05^	Bermuda	5.99 × 10^−07^
20	Pakistan	0.004811284	North Korea	0.000128562	Ghana	1.61 × 10^−05^	Antigua and Barbuda	3.52 × 10^−07^
21	Qatar	0.004113225	Mauritius	0.000126679	Armenia	1.53 × 10^−05^	Barbados	3.52 × 10^−07^
22	Macau	0.003373727	Portugal	0.000113251	Dominican Republic	1.46 × 10^−05^	Cape Verde	3.52 × 10^−07^
23	Germany	0.003176858	Uzbekistan	9.67 × 10^−05^	New Caledonia	1.34 × 10^−05^	Guyana	3.52 × 10^−07^
24	Italy	0.002753413	Hungary	8.97 × 10^−05^	Cyprus	1.29 × 10^−05^	Madagascar	2.99 × 10^−07^
25	Philippines	0.002740638	Azerbaijan	8.64 × 10^−05^	Bhutan	1.25 × 10^−05^	Grenada	2.47 × 10^−07^
26	Taiwan	0.002590034	Croatia	8.57 × 10^−05^	Nepal	1.25 × 10^−05^	Bolivia	1.76 × 10^−07^
27	Belize	0.002009996	Tajikistan	8.50 × 10^−05^	Slovenia	1.24 × 10^−05^	Burkina Faso	1.76 × 10^−07^
28	Ethiopia	0.001469205	Bahrain	6.31 × 10^−05^	Moldova	1.22 × 10^−05^	Cameroon	1.76 × 10^−07^
29	Finland	0.001307074	Fiji	6.29 × 10^−05^	Kosovo	1.21 × 10^−05^	Chad	1.76 × 10^−07^
30	Sri Lanka	0.001179859	Kyrgyzstan	6.22 × 10^−05^	Zambia	1.20 × 10^−05^	Mozambique	1.76 × 10^−07^
31	The Netherlands	0.000980799	Afghanistan	6.16 × 10^−05^	El Salvador	1.05 × 10^−05^	Paraguay	1.76 × 10^−07^
32	New Zealand	0.000971254	Panama	5.91 × 10^−05^	Romania	7.56 × 10^−06^	Solomon Islands	1.76 × 10^−07^
33	Greece	0.000958209	Morocco	5.60 × 10^−05^	Guatemala	6.92 × 10^−06^	Syria	1.76 × 10^−07^
34	Bangladesh	0.000831196	Iraq	5.54 × 10^−05^	Angola	6.77 × 10^−06^	Uruguay	1.76 × 10^−07^
35	Myanmar	0.000803755	Bulgaria	5.07 × 10^−05^	Costa Rica	6.41 × 10^−06^	Venezuela	1.76 × 10^−07^
36	Saudi Arabia	0.000750981	Lithuania	4.99 × 10^−05^	Trinidad and Tobago	6.13 × 10^−06^	Zimbabwe	1.76 × 10^−07^
37	Spain	0.000564876	Seychelles	4.82 × 10^−05^	Sudan	5.66 × 10^−06^	Libya	1.23 × 10^−07^
38	Switzerland	0.00056232	Lebanon	4.47 × 10^−05^	Ecuador	5.56 × 10^−06^	Macedonia	1.23 × 10^−07^
39	Austria	0.000498664	Georgia	4.37 × 10^−05^	Puerto Rico	4.68 × 10^−06^	Saint Lucia	1.23 × 10^−07^
40	South Africa	0.000468876	Latvia	4.06 × 10^−05^	Turkmenistan	4.42 × 10^−06^	Sierra Leone	1.23 × 10^−07^
41	Kazakhstan	0.000443175	Luxembourg	3.80 × 10^−05^	Mauritania	4.09 × 10^−06^	Somalia	1.23 × 10^−07^
42	Ireland	0.000371199	Estonia	3.69 × 10^−05^			Timor-Leste	1.23 × 10^−07^
43	Mexico	0.000322198						
44								
Number of countries/territories: 168	Africa: 2		Africa: 3		Africa: 11		Africa: 19	
	Asian: 22		Asian: 16		Asian: 4		Asian: 2	
	Pan-Europe:13		Pan-Europe: 18		Pan-Europe: 9		Pan-Europe: 4	
	North America: 4		North America: 0		North America: 3		North America: 7	
	Oceania: 2		Oceania: 2		Oceania: 6		Oceania: 3	
	South America: 0		South America: 3		South America: 8		South America: 7	
	Total: 43		Total: 42		Total: 41		Total: 42	

## Results

**Fig. 1.**
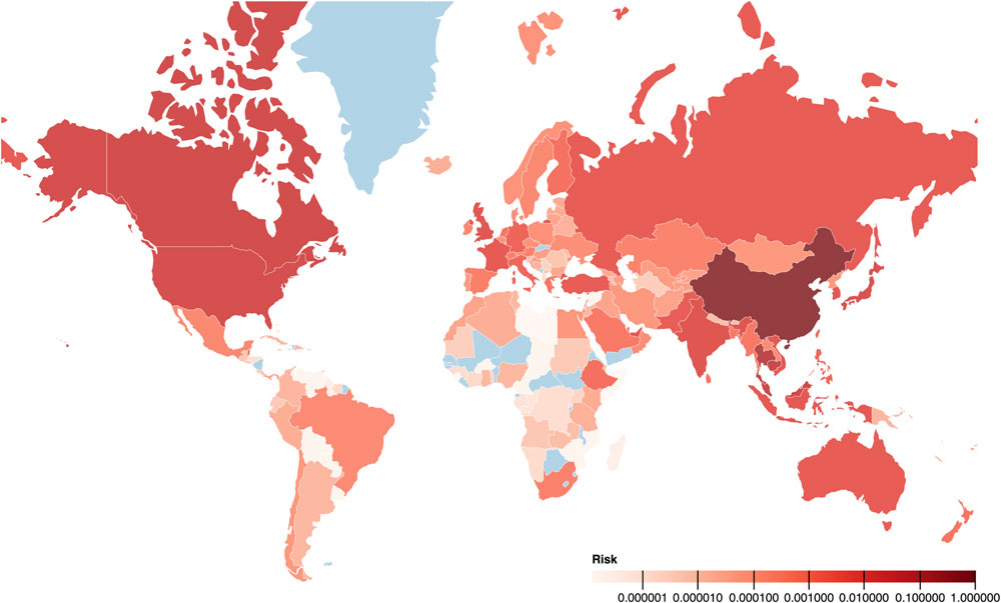
The map with the risk index of countries or territories with 2019-nCoV (SARS-COV-2) infection (0-1). The darker colour indicates higher risk and light blue colour indicates the absence of data. In general, China and neighbouring countries have a higher risk of transmission of 2019-nCoV infection. Africa and South America generally have a low risk of transmission. Ethiopia, South Africa, Egypt, Mauritius and Brazil have a similar risk of transmission to countries where at least one case has been detected. For example, the risk index of 0.1 for Thailand indicates that based on travel patterns observed during 1–31 January 2020 from four major cities of China, Thailand has 10% risk of importing a 2019-nCoV-infected person from China.

We modelled 388 287 passengers travelling to 1297 airports in 168 countries or territories. The risk index of 2019-nCoV for these countries is presented in Figure 1. A regularly updated risk map is hosted on PANDORA's website ( https://ncovdata.io/import/).

**Fig. 2.**
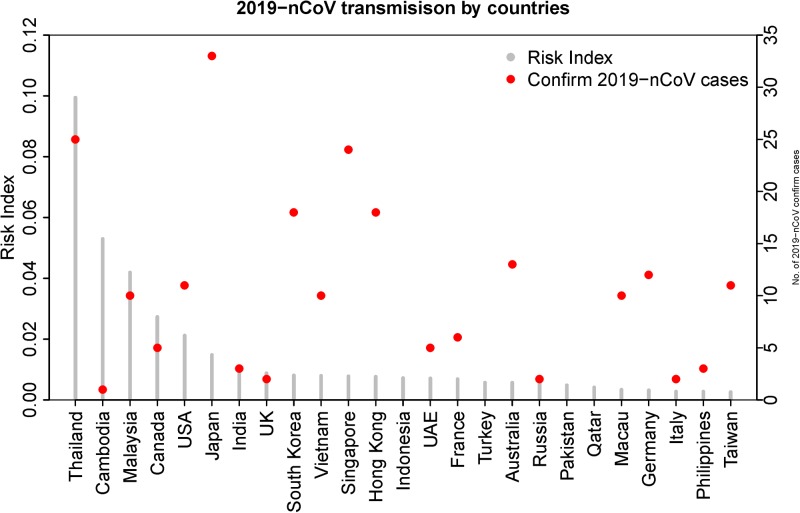
Chart showing the relative risk of countries outside China being exposed to coronavirus ( SARS-COV-2) transmission. The second *Y*-axis indicates the number of confirmed COVID-19 cases reported by the WHO as of 5 February 2020. Twenty-one of the top 25 at-risk countries (except Indonesia, Turkey, Pakistan and Qatar) reported at least one COVID-19 case by 5 February 2020.

Outside China, the countries with the highest risk of 2019-nCoV transmission from our model were Thailand, Cambodia, Malaysia, Canada and the USA, all of which have reported at least one case. Among the top 25 countries identified with the highest risk of 2019-nCoV transmission (Fig. 2), all countries except four (Indonesia, Turkey, Pakistan and Qatar) have detected at least one case as of 5 February 2020 (Table 1).

According to our risk score classification, of the countries that reported at least one 2019-nCoV case as of 5 February 2020, 24 countries were in the 4^th^ quantile of the risk index, two (Sweden and Belgium) in the 3^rd^ quantile, one (Nepal) in the 2^nd^ quantile and none in the 1^st^ quantile [14]. Asian and European countries are dominant in the 3^rd^ and 4^th^ quantile (high-risk index) while African and South American Countries are the majority in the 1^st^ and 2^nd^ quantiles (low-risk index) (Table 1). Out of 43 countries in the 4^th^ quantile, 22 were from Asia and 13 from Pan-Europe, whereas in the 1^st^ quantile, 19 out of 42 countries were from Africa (Table 1).

The overall risk of transmission of the virus into Africa is low. However, Ethiopia, South Africa, Egypt and Mauritius have a similar risk score as countries where at least one case was detected. In South America, only Brazil has a similar or greater risk than countries currently reporting cases. In North America, both the USA and Canada have high risk and had imported cases reported early in the outbreak. Australia and New Zealand have risk similar to the countries where at least one case is detected. Although there are few direct flights from China to African destinations, a large number of indirect flights operate via Dubai, an international airport hub in the United Arab Emirates.

The correlation coefficient between the estimated risk index of the countries and the WHO-reported confirmed cases was 0.97.

## Discussion

Our analysis showed a high risk of transmission of 2019-nCoV through air flights from four Chinese cities to neighbouring Asian countries. The risk of 2019-nCoV (SARS-COV-2) transmission was relatively low in Africa and South America. Several countries in both North America and Oceania showed high risk with these countries reporting at least one case of 2019-nCoV. Our risk index showed a very high correlation with the WHO's reported COVID-19 cases.

China has four times as many air passengers now than it had during SARS outbreaks in 2003. A large number of workers now travel internationally where China is heavily investing in infrastructure development in Africa, parts of Asia and Latin America. A significant and mobile Chinese population live in Europe and North America alongside an increasing amount of Chinese tourism. This travel poses a high risk of 2019-nCoV travelling across international borders. Although acquiring a case is low for these countries, the consequences are likely to be higher because of the country's capacity to control such situations [15].

Based on our model, the countries with the highest risk index but have not reported any case of 2019-nCoV as yet are Indonesia, Pakistan, Turkey, Qatar and Ethiopia. These countries are at risk and they should be the priorities for investment in case detection and airport screening. Compared to the SARS outbreak of 2003, the situation in 2020 differs due to the increased frequency and volume of international air travel.

During these early stages of the epidemic, case numbers have doubled on average every 7.4 days with an estimated basic reproduction number (*R*_0_) of 2.2 (1.4–3.9) [1]. Although the data so far suggest that the disease is mild in most cases and that the case fatality rate is currently reported to be lower than SARS or MERS, the situation is likely to go on for months and could cause severe disruption in countries that are not well prepared. Hence countries ranked as high risk in our model (4^th^ and 3^rd^ quantiles) should take all steps necessary to ensure prompt detection of cases and the capacity to manage these cases to prevent ongoing spread. International investment needs to be directed especially to countries with limited healthcare and public health surveillance capacity to enable the detection of cases and disease control [16, 17]

Our estimation showed a lower risk of transmission in Africa and South America. Nevertheless, low and middle countries on these continents are more likely to see the ongoing spread and major disruption from the introduction of a single case, even if the risk of importation is lower. Direct flights between Chinese cities and African countries are few which has contributed to a lower estimated risk of 2019-nCoV transmission. As of 5 February 2020, five cases have been reported from the United Arab Emirates (UAE) which acts as an important travel hub for onward journeys to the African continent. Implementation of mildly symptomatic passenger screening in the UAE may reduce the potential for 2019-nCoV to enter Africa. Screening and diagnostic capacity in Africa has been supported by a rapid grant from the Bill and Melinda Gates Foundation to the African CDC, mitigating the consequences of an importation.

The current situation is extremely dynamic and since then some countries have instigated flight restrictions and closed borders (e.g. Russia). These decisions were relevant for these locations but not based on probabilities. WHO has not recommended a cessation of transportation to free countries but suggested preventive measures. This would seem appropriate for Africa and South America with the caveat that only one case is needed to initiate a local epidemic without proper biosecurity and quarantine measures, whilst other regions will need to decide on a case-by-case basis through appropriate risk assessment.

Our study has several limitations. We considered flights from four cities of China, three of which (Beijing, Shanghai and Guangzhou) are ranked among top five busiest airports (based on the number of flights) in China and Wuhan as the site of origin of outbreaks. While including further cities in our analysis would have added further information, Beijing and Shanghai cover most of the international destinations to which other airports are connected. Further, we have adjusted for the number of reported cases in each departure. When developing the model, we initially explored using only Wuhan as the departure airport, the rank of top 10 at-risk countries remained the same. Thus our findings are still representative of the total risk posed by other airports or cities. We did not consider the risk associated with the travel route through water and land which might have an impact in the spread of 2019-nCoV. Another limitation is that the model does not account for travel patterns in other affected countries. For example, some cases have started acquiring the disease outside of China: the third case notified in the UK acquired the disease in Singapore. However overall, the risk compared with the risk of acquisition in China is very low, therefore it probably would not change the order of countries.

## Conclusion

The risk of transmission of SARS-COV-2 from China on 31 January 2020 was highest to neighbouring countries in Asia (Thailand, Cambodia, Malaysia), followed by Europe (UK, France, Russia and Germany), Oceania (Australia and New Zealand) and North America (USA and Canada). The situation is dynamic and may have changed with the closure of flights and borders since this analysis was done. The higher correlation coefficient with travellers and case detection data indicate that 2019-nCoV will remain a significant threat from the air-borne movement of people. The authors suggest an ongoing risk-based approach to the prioritisation of and investment by international and national agencies and authorities, in emergency interventions for the prevention of movement of 2019-nCoV (SARS-COV-2) through human travel. This is achieved by appropriate actions at high-risk points of departure and at highly used ports of entry from these infected zones. Closure of certain routes, targeted airport screening, risk communication, public awareness and targeted training and vigilance of health workers associated with the portals of entry of visitors to their countries will help mitigate the force of further spread of 2019-nCoV.
